# Effectiveness of Social Media-Based Intervention in Intention Change of Adolescents for Promoting Sexual Health Behavior in Western Terai of Nepal

**DOI:** 10.34172/jrhs.2024.148

**Published:** 2024-06-01

**Authors:** Ganesh Bhandari, Shalik Ram Dhital, Dipika Khatri, Tulsi Ram Bhandari

**Affiliations:** ^1^School of Health, Allied Sciences, Pokhara University, Pokhara, Nepal; ^2^Nepal Health Professional Council, Kathmandu, Nepal

**Keywords:** Adolescent, Intention, Sexual health, Intervention, Health promotion, Social media, Nepal

## Abstract

**Background:** Sexually transmitted infections (STIs) in adolescents are among the major public health challenges that have to be prevented in time. Traditional education falls short of reach; social media offers accessible ways. However, there is no research on such an issue in Nepal. Accordingly, this study was conducted to assess the effectiveness of a social media-based health education intervention in changing the intention of promoting sexual health among adolescents in Nepal.

**Study Design:** A quasi-experimental study.

**Methods:** A total of 160 adolescent students aged 14–19 years old from four purposively selected schools were evenly divided into intervention and non-intervention groups. Sampling and data collection were performed between May and June 2023. Data were collected through self-administered questionnaires for pretest and posttest evaluation. The intervention was delivered and followed up through a Facebook Messenger group. The obtained data were managed and analyzed using SPSS 21, with a significance level of 5%.

**Results:** Social media-based health education interventions played a significant role in promoting the sexual health behavior of adolescents. The adolescents’ knowledge and attitude scores on STIs increased from 2.33 to 4.62 and from 21.87 to 26.30. In addition, their scores on subjective norms, perceived behavioral control, and intentions in promoting sexual behavior increased from 13.93 to 17.59, from 19.96 to 25.40, and from 13.07 to 18.06, respectively, which were statistically significant.

**Conclusion:** The utilization of social media platforms such as Facebook Messenger groups is an effective medium for delivering health educational messages. Hence, increasing social media-based health education is a cost-effective intervention for promoting the health and sexual behaviors of adolescents.

## Background

 A sexually transmitted infection (STI) is an infectious disease primarily transmitted through unprotected sexual contact and, in some cases, during pregnancy, childbirth, breastfeeding, or through infected blood, with potentially serious health consequences and a range of associated social and reproductive impacts.^1^ The adolescence age group of 10–19 years is a critical phase of development characterized by physical, cognitive, and emotional changes. In this stage of life, adolescents face significant challenges such as sexual and reproductive health behaviors, making it a vital focus for health promotion interventions. They can face problems such as mental health, teenage pregnancy, STIs, violence, and the like.^2^ A central aspect is the lack of access to adequate and correct information about sexual and reproductive health, leading to risky sexual behavior among adolescents.^3^ Misinformation and limited sexual health cause unhealthy behavior and increase the risk of infection.^4^

 While efforts have been made to address adolescents’ sexual health, traditional health education approaches may not be fully effective in reaching and engaging adolescents. However, the increasing popularity of social media platforms among adolescents offers a promising avenue for health promotion.^4^ Media platforms such as Facebook and Messenger, Instagram, and Twitter offer widespread accessibility and feasibility. Despite these potential benefits, research on social media and their effects on adolescents’ sexual health remains limited in Nepal.^5,6^ Existing studies have primarily focused on the broader use of social media for health promotion rather than targeting adolescents’ sexual health specifically. Therefore, this paper assessed the effectiveness of a social media-based health education intervention in changing the intention of promoting sexual health among adolescents in Nepal.

## Methods

###  Study setting and population 

 This study was conducted in Suklaphanta Municipality, Nepal. Students aged between 14 and 19 years old from four different higher secondary schools were study participants and were selected if they used social media and had internet access.

###  Study design

 This quasi-experimental study used a pretest and posttest control group design. The total sample size for both groups was 158, with a 1:1 ratio between them. This sample size was obtained after adding 20% attrition to the calculated size of 126.

 The sample size is calculated using the online platform Openepi (*https://www.openepi.com/SampleSize/SS.htm*), with the following statistical details:

 Two-sided significance level (1-alpha) = 95%

 Power (1-beta) = 80%

 Ratio of sample size, unexposed/exposed = 1:1

 Prevalence difference = 19% ^7^

 It is important to note that a total of 160 samples were collected for this research due to the complete enumeration.

###  Measures 

 The primary outcome measure was the participants’ behavioral intention, which was assessed through a self-administered questionnaire before and after the intervention. The questionnaire was developed based on the key constructs of the theory of planned behavior (TPB), including attitudes, subjective norms, and perceived behavioral control. The measurement scales were knowledge (ranging from 0 to 7), attitude (ranging from 6 to 30), subjective norm (ranging from 4 to 20), perceived behavioral control (ranging from 6 to 30), and intention (ranging from 4 to 20).

 To ensure the validity and reliability of the questionnaire, a pretest was conducted, and necessary modifications were made based on participants’ feedback. The internal consistency of the tools was assessed using Cronbach’s alpha. Data were collected through a complete enumeration of the selected school students who met the inclusion criteria, and the intervention group received a social media-based health education intervention package, including educational materials, infographics, quizzes, e-posters, and interactive activities. The non-intervention group received only basic educational materials.

###  Intervention

 The intervention was delivered through a Facebook Messenger group. The intervention package was carefully developed based on a review of existing literature, formative research, and the TPB constructs.

 The content of the intervention package included a variety of health education materials comprised of infographics, quizzes, e-posters, fun facts, and interactive online discussion activities. These materials were presented in a culturally appropriate and language-friendly manner to ensure easy understanding and engagement. The face validity of the package was ensured by obtaining feedback from external reviewers. Content validity was confirmed through expert consultation, ensuring that the materials effectively explained the topics and were user-friendly media for the target audience.

**Figure 1:**
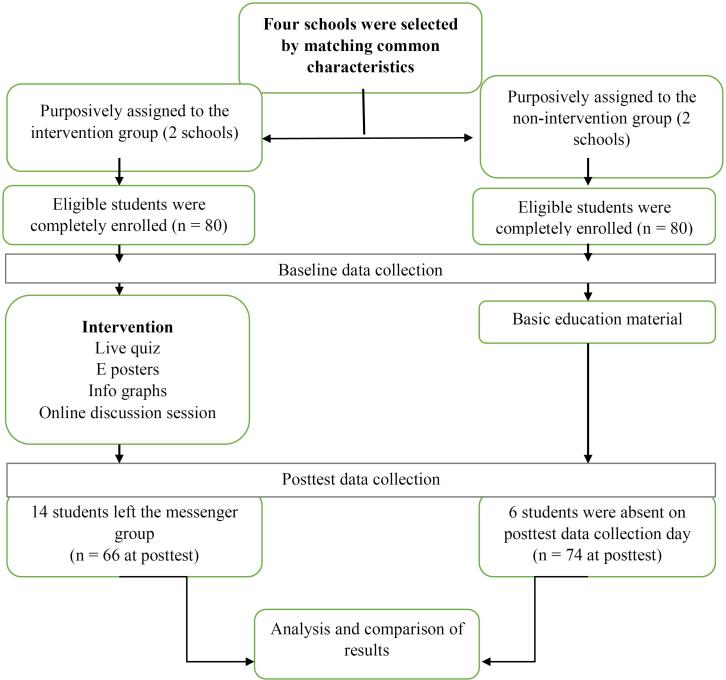
Study Flow Diagram

 During the 15-day intervention, participants in the intervention group were actively engaged through the Facebook Messenger group. They were invited to participate in quizzes to test their knowledge of sexual health topics, followed by engaging them in the discussion session related to STI prevention and safer sexual behavior (Figure 1).

###  Statistical analysis

 Descriptive statistics were presented in the form of frequencies, percentages, and means. Inferential statistics such as paired t-tests assessed changes within groups, while independent t-tests were used to compare intervention and non-intervention outcomes post-intervention within a 5% level of significance. Correlations and multiple linear regression analyzed the relationships between TPB constructs and identified predictors for the intention of promoting sexual behavior. The statistical analysis was conducted using SPSS software, version 21.

###  Public and patient involvement

 Throughout the study, adolescent students and teachers were actively engaged to ensure the relevance and effectiveness of our research in promoting sexual health behavior among adolescents in Suklaphanta Municipality, Nepal.

 Prior to initiating the study, we held focus-group discussions and consultations with adolescents, teachers, and the municipality education section officer from the study population. Their input and feedback were instrumental in shaping the study design, intervention strategies, and data collection tools. They also played a vital role in the development of the social media-based intervention package. They provided insights into the content, language, and cultural aspects to ensure that the materials were accessible and engaging. External reviewers and expert consultations further validated the intervention’s content and format.

 Additionally, we acknowledged the significance of ethical issues and made sure that the research procedure safeguards the participants’ confidentiality and privacy. The study’s design and objectives were made known to guardians, and parental approval was obtained because some of the adolescents were under the age of 18 at the time.

 By involving members of the public, specifically adolescents, and the local community in these ways, it was aimed to co-produce research that genuinely addressed the concerns and preferences of the population in Suklaphanta Municipality.

## Results

###  Socio-demographic characteristics

 This study included 160 participants, with 80 in the intervention group and 80 in the non-intervention group. The mean age of participants was similar in both groups, with most falling within the age range of 14–19 years. The majority of participants in the intervention and non-intervention groups were male. In both groups, Brahmin/Chhetri were the most common caste/ethnicity. Most parents in both groups were married, and nuclear families were predominant. Non-formal education stands out as a common educational background among mothers in both groups. Agriculture was the primary occupation for both mothers and fathers. The most used social media platform was Facebook. Moreover, the majority of participants in both groups used mobile data for internet access. The wealth index indicated a relatively even distribution between rich and poor participants.

###  Response to the intervention 

 At baseline, only 10% of participants in the intervention group reported receiving any STI prevention message previously through social media. However, after the intervention, more than 90% of participants in the intervention group reported receiving STI prevention messages, indicating a significant increase in exposure to such messages. Among those who received STI prevention messages, the majority (95%) accessed them through Facebook Messenger. Moreover, the health education intervention had positive effects on participants, with 68% mentioning its impact, such as motivating them to practice safe sex (26%), improving communication about sexual health with partners (24.5%), and dispelling myths and misconceptions about STIs (16.5%).

###  Impact on knowledge and behavior 

 In the intervention group, there were statistically significant improvements in knowledge, attitude, subjective norms, perceived behavioral control, and intention scores at the posttest (Table 1).

**Table 1:** Mean scores of knowledge and theory of planned behavior constructs (within groups)

**Variables**	**Pretest**		**Posttest**		**Mean difference**	**t**	***P*** ** value**
	**Mean**	**SD**	**Mean**	**SD**			
**Non-intervention group**							
Knowledge	2.14	1.36	2.10	1.39	0.04	0.30	0.760
Attitude	22.31	2.34	20.12	1.90	2.18	6.87	0.001
Subjective norms	14.50	2.10	14.67	2.09	-1.70	-0.49	0.630
Perceived behavioral control	20.94	4.26	21.12	4.32	-1.70	-0.23	0.820
Intention	13.79	3.10	12.35	1.80	1.44	3.30	0.001
**Intervention group**							
Knowledge	2.33	1.41	4.62	1.56	-2.38	-10.71	0.001
Attitude	21.87	3.25	26.39	4.33	-4.51	-6.40	0.001
Subjective norms	13.93	2.29	17.59	2.23	-3.65	-8.59	0.001
Perceived behavioral control	19.96	4.13	25.40	4.41	-5.43	-7.31	0.001
Intention	13.07	3.26	18.06	3.02	-4.98	-8.65	0.001

*Note*. SD: Standard deviation.

 The intervention group exhibited a significantly higher mean knowledge score compared to the non-intervention group. The difference is statistically significant (t = 10.05, *P* < 0.001). Similarly, there were significant differences in mean attitude scores, subjective norms, perceived behavioral control scores, and intention scores between both groups at the posttest (Table 2).

**Table 2:** Knowledge scores between non-intervention and intervention groups at posttest (between groups)

**Variables**	**Non-intervention group**		**Intervention group**		**t**	***P*** ** value**
	**Mean**	**SD**	**Mean**	**SD**		
Knowledge	2.10	1.39	4.62	1.56	10.05	0.001
Attitude	20.12	1.90	26.39	4.33	11.29	0.001
Subjective norms	14.67	2.09	17.59	2.23	138.00	0.001
Perceived behavioral control	21.12	4.32	25.40	4.41	138.00	0.001
Intention	12.35	1.80	18.06	3.02	138.00	0.001

*Note*. SD: Standard deviation.

###  Correlation with the theory of planned behavior 

 The findings demonstrated that in both the non-intervention and intervention groups, attitude, subjective norm, and perceived behavioral control had positive relationships to changing behavior related to STI prevention. These relationships were consistently observed at both baseline and posttest. However, in the intervention group, the correlations between these factors and intention were stronger at the posttest compared to the baseline, indicating that the intervention likely strengthened the influence of these psychological factors on participants’ intentions. In contrast, the influence of subjective norms on intention weakened in the non-intervention group in the posttest (Table 3).

**Table 3:** Correlation Between Theory of Planned Behavior Constructs at Baseline and Posttest

**Variables**	**Non-intervention Group**				**Intervention Group**			
	**Attitude**	**Subjective** **Norms**	**Perceived** **Behavior Control**	**Intention**	**Attitude**	**Subjective** **Norms**	**Perceived** **Behavior Control**	**Intention**
**Pretest**								
Attitude	1.00				1.00			
Subjective norm	0.37	1.00			0.52	1.00		
Perceived behavioral control	0.22	0.43	1.00		0.40	0.44	1.00	
Intention	0.26	0.29	0.49	1.00	0.40	0.41	0.61	1.00
**Posttest**								
Attitude	1.00				1.00			
Subjective norm	0.47	1.00			0.52	1.00		
Perceived behavioral control	0.01	0.40	1.00		0.62	0.73	1.00	
Intention	0.27	0.15	0.17	1.00	0.61	0.32	0.47	1.00

###  Predictor of intention for behavior change

 The study findings showed attitude and perceived behavioral control as significant predictors of the intention to change behavior related to STI prevention. For every unit increase in attitude and perceived behavioral control, the intention to change behavior was predicted to increase by 0.372 units. Attitude explains about 46.8% of the unique variance in intention, while perceived behavioral control accounts for 18.5% of the unique variance (Table 4).

**Table 4:** Predictor for Intention to Behavior Change (Δ R^2^ = 0.398)

**Constructs**	**B**	**SE**	**β**	**Partial R**^2^
Attitude	0.372	0.080	0.533	0.468
Subjective norm	-0.170	0.190	-0.126	-0.109
Perceived behavior changes	0.372	0.109	0.236	0.185

*Note*. SE: Standard error.

## Discussion

 This paper evaluated the effectiveness of a social media-based health education intervention in changing the intention of promoting sexual health among adolescents in Nepal. The findings of this study confirmed the potential of such health education interventions to bring significant changes in participants’ knowledge, attitudes, subjective norms, perceived behavioral control, and intentions related to STI prevention.

 Comparing the baseline knowledge scores of both the non-intervention and intervention groups revealed a common lack of awareness regarding STI prevention. This corresponds to previous studies focusing on human immunodeficiency virus/acquired immunodeficiency syndrome (HIV/AIDS) prevention, highlighting the need for targeted interventions to enhance knowledge and awareness. These findings align with those of some studies, implying that educational interventions can effectively improve knowledge and awareness regarding STI and HIV/AIDS transmission.^8,9^

 The intervention group exhibited substantial improvements in attitudes, subjective norms, perceived behavioral control, and intentions compared to the non-intervention group. The positive impact of the intervention on attitudes toward STI prevention conforms to the findings of similar research in Southwest Ethiopia, which also emphasized positive attitudes toward preventive measures^8^. The intervention also effectively influenced participants’ perceptions of social norms, potentially fostering a sense of social support and encouragement for adopting preventive behaviors.

 The intervention positively influenced participants’ perceived behavioral control over engaging in STI prevention behaviors. This outcome is in line with the results of research from Tehran, where perceived behavioral control significantly improved following an educational intervention.^10^ Furthermore, current health education interventions significantly enhanced participants’ intentions to change behavior, which is consistent with the findings of a study conducted in Iran, demonstrating higher improvements in behavioral intention after educational interventions.^11^

 A significant relationship was observed between attitude, perceived behavioral control, and intention to change behavior both before and after the intervention. Positive attitudes were associated with stronger intentions, highlighting the role of attitudes in driving behavioral change. Perceived behavioral control consistently influenced intention, underscoring the importance of individuals’ confidence in their ability to engage in preventive behaviors. These findings corroborate those of another study utilizing theoretical frameworks such as the TPB.^12^

 The findings of this study indicated that attitude plays a significant role in shaping individuals’ intentions to change their behavior. Positive attitudes toward STI prevention were associated with stronger intentions to engage in preventive behaviors. Attitude explained a substantial amount of the variance in intention, emphasizing its importance in driving behavior change. However, subjective norms had no significant effect on intention in this study. This finding contradicts the findings of previous research that identified subjective norms as a significant predictor of condom use and intention^13^. On the other hand, perceived behavioral control was found to have a significant and positive effect on intention, which aligns with previous studies that have consistently demonstrated the influence of perceived behavioral control on condom use and intention.^14-17^ Interventions for increasing perceived behavioral control are effective in promoting condom use.^15,17,18^ The findings of this study are supported by a body of research, confirming the importance of attitude and perceived behavioral control in predicting condom use and intention.^14-18^ Various studies have reported that these factors significantly influence individuals’ decision-making regarding condom use. Interventions targeting attitude and perceived behavioral control have shown promise in promoting condom use and safer sexual practices.^15,16,18^ This intervention, which utilized a Facebook Messenger group for information sharing and interactive sessions, showcases the potential of technology-driven interventions to impact attitudes, subjective norms, and perceived behavior control effectively. This aligns with the findings of similar studies that leveraged mobile technologies and social media platforms to promote health-related behaviors.^19^

 This finding suggests that social media-based health education interventions hold promise for promoting positive sexual health behaviors among adolescents in schools. Accordingly, it is suggested that future research explore the long-term effects and sustainability of such interventions, examine different social media platforms, and consider qualitative methods for deeper insights into participants’ experiences. Additionally, investigating external influences and their interactions with social media interventions will provide a more comprehensive understanding of their effects. The long-term consequences of interventions on behavioral intentions and behavior change should also be a priority for future research.

 Firstly, this study recognizes response bias due to the data being self-reported and recommends utilizing extra indicators for validation. Secondly, it is hard to generalize the results due to short-term intervention, and therefore further long-term intervention studies remain to be performed in this regard. Finally, it was hard to say which methods and media worked well for change.

**Highlights**

- **Effective 15-Day Facebook Messenger Intervention:** It improved adolescents’ knowledge, attitudes, norms, behavioral control, and intentions regarding sexual health.
- **Noteworthy Improvements Post-Intervention:** It could enhance scores on STI knowledge, positive attitudes, social norms, behavioral control, and intentions for promoting sexual health.
- **Cost-Effective Social Media Promotion:** Facebook Messenger groups were proven to be cost-effective for adolescent health education, showcasing digital tools’ potential in addressing sexual health challenges.

## Conclusion

 The findings revealed the power of social media-based health education interventions in promoting positive intentions toward behavior change related to STI prevention and care. Participants’ knowledge, attitudes, and intentions were positively influenced through engaging content on platforms such as Facebook Messenger, highlighting the potential of digital tools for promoting health and addressing STI-related challenges in youth.

## Acknowledgments

 The authors acknowledge the Institution Ethical Review Committee of Pokhara University for ethical approval. Similarly, our sincere gratitude goes to the study participants and their parents for their support and cooperation.

## Authors’ Contribution

**Conceptualization:** Ganesh Bhandari, Tulsi Ram Bhandari.

**Data curation:** Ganesh Bhandari, Dipika Khatri.

**Formal analysis:** Ganesh Bhandari, Dipika Khatri.

**Funding acquisition:** Ganesh Bhandari, Dipika Khatri.

**Investigation:** Ganesh Bhandari, Tulsi Ram Bhandari.

**Methodology:** Ganesh Bhandari, Tulsi Ram Bhandari.

**Project administration:** Ganesh Bhandari, Tulsi Ram Bhandari.

**Resources:** Ganesh Bhandari, Shalik Ram Dhital.

**Software:** Ganesh Bhandari.

**Supervision:** Shalik Ram Dhital, Tulsi Ram Bhandari.

**Validation:** Tulsi Ram Bhandari.

**Visualization:** Ganesh Bhandari, Tulsi Ram Bhandari.

**Writing–original draft:** Ganesh Bhandari, Shalik Ram Dhital, Tulsi Ram Bhandari.

**Writing–review & editing:** Ganesh Bhandari.

## Competing Interests

 The authors declare that they have no competing interests.

## Ethical Approval

 Ethical approval was obtained from the Institutional Review Committee under Pokhara University’s (reference No. 032-079/80), Kaski, Nepal. This study followed ethical guidelines and obtained informed consent from the study participants and their parents. Confidentiality was maintained, and participants’ well-being was prioritized throughout the research process.

## Funding

 This study received no financial support.
